# Platelets and the complement cascade in atherosclerosis

**DOI:** 10.3389/fphys.2015.00049

**Published:** 2015-03-02

**Authors:** Johannes Patzelt, Admar Verschoor, Harald F. Langer

**Affiliations:** ^1^University Clinic for Cardiovascular Medicine, University of TuebingenTuebingen, Germany; ^2^Institute for Medical Microbiology, Immunology and Hygiene, Technische Universität MünchenMunich, Germany; ^3^Section for Cardioimmunology, Department of Cardiovascular Medicine, University of TuebingenTuebingen, Germany

**Keywords:** atherosclerosis, platelets, complement system proteins, innate immunity, inflammation

## Abstract

Atherosclerosis and its late sequels are still the number one cause of death in western societies. Platelets are a driving force not only during the genesis of atherosclerosis, but especially in its late stages, as evidenced by complications such as arterial thrombosis, myocardial infarction, and ischemic stroke. Atherosclerosis is increasingly recognized as an inflammatory disease, influenced by various immune mechanisms. The complement system is part of our innate immune system, and its diverse roles in atherosclerosis have become evident over the past years. In this review we identify points of intersection between platelets and the complement system and discuss their relevance for atherosclerosis. Specifically, we will focus on roles for platelets in the onset as well as progression of the disease, a possible dual role for complement in the genesis and development of atherosclerosis, and review emerging literature revealing previously unrecognized cross-talk between platelets and the complement system and discuss its possible impact for atherosclerosis. Finally, we identify limitations of current research approaches and discuss perspectives of complement modulation in the control of the disease.

## Introduction

Despite remarkable progress in therapy, atherosclerosis and its associated complications such as coronary heart disease are still the leading cause of death in western societies (Ross, 1999). Atherosclerosis is defined as the process of plaque formation and stenosis in arterial vessels. Deleterious effects of plaque rupture include acute vessel occlusion and thromboembolic complications such as myocardial infarction resulting from thrombotic occlusion of an epicardial vessel or stroke resulting from carotid artery plaque formation.

**Figure 1:**
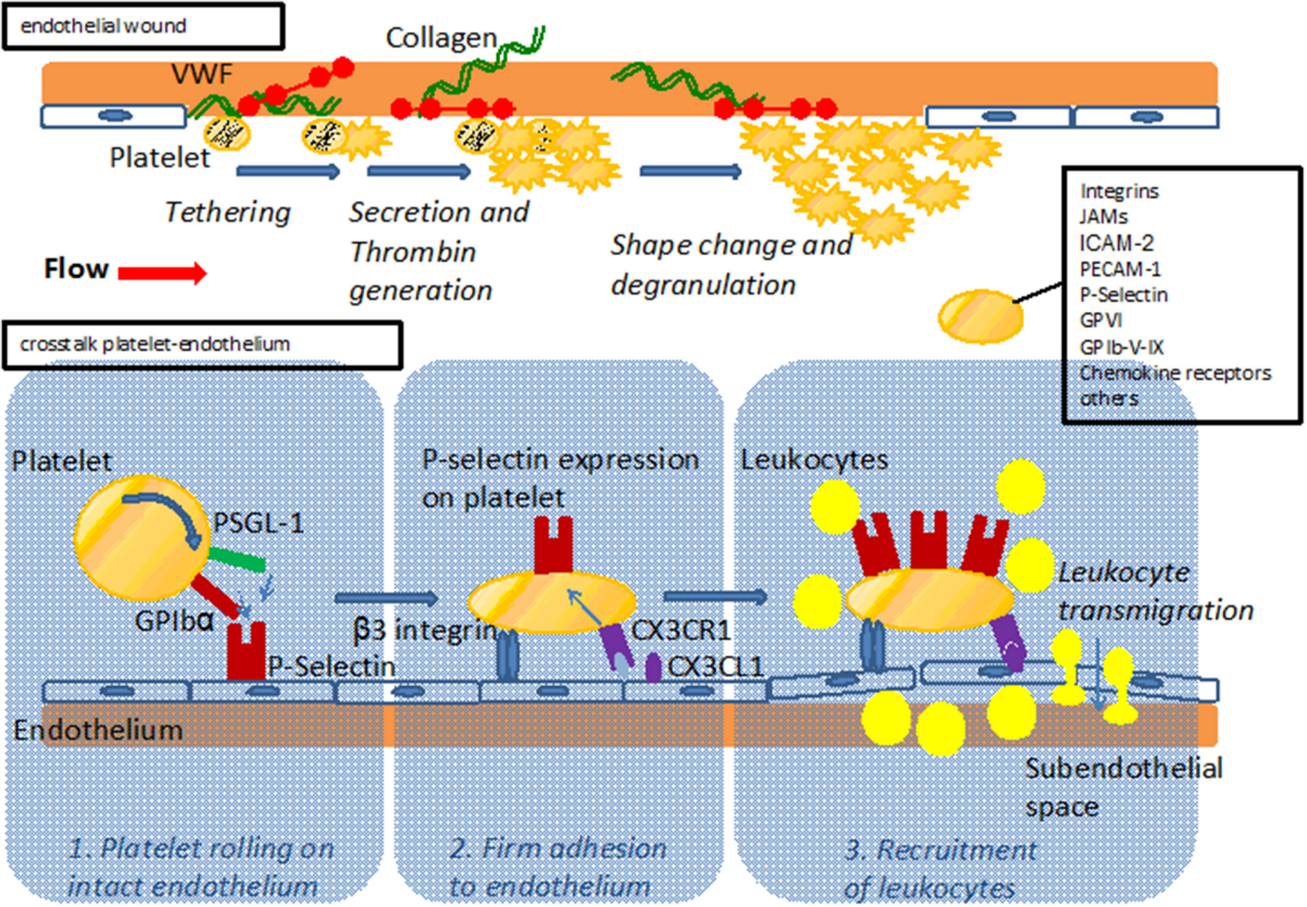
**Upper part:** Model of platelet interaction with the damaged vessel wall: exposure of subendothelial matrix after endothelial lesion leads to platelet tethering, activation and accumulation to provide sealing of the endothelial wound. **Lower part:** Model of platelet interaction with the endothelium. Platelets interact with the endothelium via adhesion receptors such as GPIbα and PSGL-1 promoting rolling and subsequent firm adhesion via β3 integrins. Interaction with endothelial bound chemokines such as Cx3CL1 (fractalkine) or CCL5 (Rantes) results in P-Selectin mediated recruitment of leukocytes to the vessel wall and subsequent transmigration. For both interaction with the subendothelial matix and the endothelium, major adhesion receptors expressed on platelets are listed.

A complex interplay between hemostatic and inflammatory cues is increasingly unraveled and recognized to drive the genesis and exacerbations of atherosclerosis. Platelets are thought to play a dual role here, mediating the often serious clinical effects of plaque rupture in established atherosclerosis, but also impacting the early development of atherosclerosis (Massberg et al., 2002). The classical function of platelets is coverage and closure of endothelial wounds, and contact between platelets and the subendothelial matrix triggers their activation and drives thrombus formation also during the early pathophysiological process of plaque formation (Figure 1). Moreover, platelets have been shown to also interact with intact endothelium and recruit leukocytes even before an atherosclerotic plaque has formed (Massberg et al., 2002).

On the other hand, atherosclerosis is recognized as an inflammatory disease (Ross, 1999), making the immune system a focus of interest. The complement system, as part of our immune system, has important protective functions in immune defense but can also be a driving force behind the pathological processes of chronic inflammatory diseases (Walport, 2001). The complement system consists of a series of plasmatic proteins (zymogens) that form enzymatic cascades, which produce a broad spectrum of immune-active molecules and pro-inflammatory mediators (Verschoor and Carroll, 2004). Besides these classical functions of immune defense, the complement system also directs central homeostatic and pathophysiological processes such as angiogenesis, tissue regeneration, the removal of immune complexes, apoptotic cells, and cellular debris (Ricklin et al., 2010). Thus, with respect to atherosclerosis complement may exert proatherogenic as well as atheroprotective effects, for which we will discuss the evidence below in detail.

## Platelets and atherosclerosis

Platelets have a well-recognized contribution to the late thrombotic complications associated with atherosclerosis, making them a potent target for the development of diagnostic and therapeutic tools (Gawaz et al., 2008). In addition, platelets can also interact with endothelial cells before an atherosclerotic plaque has formed and may thus play a role in the genesis of atherosclerosis (Gawaz et al., 2005). Indeed, platelets of atherosclerotic apolipoprotein E deficient (ApoE–/–) mice were observed to adhere to intact endothelium via the von Willebrand factor receptor GPIbα and the fibrinogen receptor GP_IIbIIIa_ well before atherosclerotic plaques had formed (Massberg et al., 2002; Huo et al., 2003; Gawaz et al., 2005). Still, absence of GP_IIbIIIa_ alone does not fully protect from atherosclerosis, as is known from studies assessing patients with Glanzmann thrombasthenia whose platelets lack functional GP_IIbIIIa_. Indeed, ultrasound imaging of the carotid bifurcation of such patients revealed plaques in 4 of 7 individuals (Shpilberg et al., 2002). Therefore, platelet-vessel wall interactions via GP_IIbIIIa_ may contribute to, but do not seem to be a prerequisite in the genesis of human atherosclerosis and may be functionally substituted by other platelet receptors. Mice with deficiency in GPIbα are not protected from atherosclerotic plaque formation (Strassel et al., 2009). On the other hand, platelet depletion with a GPIbα-specifc antibody in ApoE–/– mice leads to reduced leukocyte accumulation in the arterial intima and attenuated atherosclerotic plaque formation, importantly indicating that adhering platelets form a focal point for the immune-cell driven inflammation that typifies atherosclerosis (Massberg et al., 2002). Indeed, PSGL-1 or GPIbα allow platelets to interact with P-selectin expressed on intact endothelium. This initial “rolling” interaction is followed by β3 integrin–mediated firm “adhesion” of platelets to the vessel wall (Gawaz et al., 2005), and these events are seen as crucial steps in the initiation of atherosclerosis. Platelets express various inflammatory receptors, including fractalkine receptor (CX3CR1) which induces P-selectin on platelets upon binding to fractalkine (CX3CL1) expressed on inflamed endothelial cells (Schulz et al., 2007). In turn, P-selectin exposure initiates local accumulation of leukocytes, driven by arterial shear forces (Schulz et al., 2007) (Figure 1). The importance of P-selectin in the genesis of atherosclerosis is underlined by the finding of increased intima-media-thickness in human subjects presenting with high levels of platelet P-selectin (Koyama et al., 2003). Indeed, both platelet and endothelial P-selectin contributed to lesion formation in a mouse model of atherosclerosis that was based on the adoptive transfer of P-selectin positive or negative platelets (Burger and Wagner, 2003).

Whether platelet-adhesion to the intima mediates direct damage to the endothelial lining remains unclear, but several studies found how platelets contribute to vascular inflammation via their interaction with leukocytes (Santoso et al., 2002; Schober et al., 2002; Langer et al., 2007, 2012; Ley et al., 2007; Langer and Chavakis, 2009). Activated platelets were shown to exacerbate atherosclerosis in ApoE deficient mice via the recruitment of monocytes and other leukocytes (Wagner and Frenette, 2008; von Hundelshausen et al., 2009). In turn, the formation of platelet-leukocyte aggregates (PLA) facilitated the deposition of inflammatory platelet mediators on endothelial cells (Schober et al., 2002; Huo et al., 2003). Others found that the number of circulating PLAs is increased upon platelet activation (van Gils et al., 2009; Totani and Evangelista, 2010). As potential mechanisms underlying platelet-leukocyte crosstalk, several receptor/ligand pairs have been identified, including integrins or members of the JAM family of proteins (von Hundelshausen and Weber, 2007; Wagner and Frenette, 2008; von Hundelshausen et al., 2009). A list of platelet expressed receptors with potential relevance for artherosclerosis is given in Table 1. Finally, platelets may also contribute to vascular inflammation via release of active biomolecules from their granules (Langer and Gawaz, 2008; Patzelt and Langer, 2012). As prominent examples, the release of chemokines such as CCL5 or CXCL4 contributes to atherosclerosis in a P-selectin dependent manner (von Hundelshausen and Schmitt, 2014).

**Table 1:** **Platelet receptors, their ligands, and their target structure contributing to vascular inflammation**.

**Receptor**	**Ligand**	**Interaction with**
PSGL-1	P-Selectin	Endothelial cells (Frenette et al., 2000)
P-Selectin	PSGL-1	Leukocytes (Dole et al., 2007)
GPIbα	Mac 1 P-Selectin	Leukocytes (Gawaz et al., 1997) Endothelial cells (Massberg et al., 2002)
GPIIb-IIIa (α_IIb_β_3_)	Mac 1 Fibrinogen, vWF	Leukocytes (Weber and Springer, 1997) Vessel wall (Bombeli et al., 1998)
α_5_β_1_, α_6_β_1_	Subendothelial extracellular matrix	Damaged vessel wall (Gruner et al., 2003)
α_2_β_1_	Collagen	Damaged vessel wall (Inoue et al., 2003)
α_V_β_3_	Vitronectin	Endothelial cells (Gawaz et al., 1997)
CX3CR1	CX3CL1	Endothelial cells (Schulz et al., 2007)
ICAM-2	LFA-1	Leukocytes(Weber et al., 2004)
JAM-A	JAM-A	Vessel wall (Karshovska et al., 2015)
JAM-C	MAC-1	Dendritic cells (Langer et al., 2007)
GPVI	Collagen	Damaged vessel wall (Massberg et al., 2003)

Given the relevance of fatty acids in the genesis of artherosclerosis, it should be noted that also oxidized LDL—one of the major initiators and drivers of atherosclerosis—can be bound by platelets and interactions with lipoproteins can change platelet function (Siegel-Axel et al., 2008; Stellos et al., 2012). In line with this, platelets of hypercholesterolemic patients show hyperaggregability *in vitro* and enhanced activity *in vivo* (Cipollone et al., 2002; Ferroni et al., 2006). In conclusion, platelet activation seems to confer proatherosclerotic effects, as well as effects of atheromodulation and tissue/vascular remodeling.

## The complement system and atherosclerosis

As noted earlier, many cells and molecular mediators that were identified to modulate the development of artherosclerosis are components of the immune system. Complement, as part of the innate immune system, has a broad range of immune-modulatory effects, including the opsonization of microbial intruders with C1q or manose binding lectin (MBL), followed by the activation products of C2, C3, and C4 (including the opsonins C3b and C4b), the induction of mast cell degranulation via soluble anaphylatoxins C3a and C5a and the attraction of inflammatory cells (Verschoor and Carroll, 2004). Components C5b–C9 form the membrane attack complex (MAC), which mediates lysis of target cells. Beyond immune defense, the complement system directs central homeostatic and pathophysiological processes in tissue remodeling and the removal of immune complexes, apoptotic cells and cellular debris (Ricklin et al., 2010). Components of the classical pathway, including C1q, C2, and C4, are associated with the homeostatic control of such complexes, as their deficiency predisposes to diseases characterized by an impairment in the removal of cellular remnants, for example known in Systemic Lupus Erythomatosis (SLE) (Aggarwal et al., 2010). Apoptotic and necrotic cells also accumulate in atherosclerotic plaques, and a Swedish study identified a significant association between genetic C2 deficiency and atherosclerosis, including a higher rate of myocardial infarctions and stroke in a cohort of 40 patients (Jonsson et al., 2005). Furthermore, in patients with the premature atherosclerotic peripheral vascular disease C4 deficiency, a significant proportion of diseased patients revealed circulating immune complexes and their enhanced propensity to immune complex formation was associated with a higher prevalence of circulating immune complexes in atherosclerotic patients (Nityanand et al., 1999). A genome-wide analysis found a SNP of C1q receptor C1qRp (CD93) constituting a risk factor for coronary artery disease, which could be confirmed in patients with familial hypercholesteremia (van der Net et al., 2008) and polymorphisms for mannose binding lectin (MBL) with decreased levels of the protein went along with more coronary artery disease and increased carotid plaque (Madsen et al., 1998; Hegele et al., 2000; Best et al., 2004).

Complement activation also promotes inflammation, through the generation of anaphylatoxins. Analyzing circulating levels of such complement components demonstrated that patients with advanced atherosclerosis present with elevated levels of anaphylatoxin C5a, predictive of major cardiovascular events and independent of known risk markers such as C reactive protein (CRP) or fibrinogen (Speidl et al., 2005).

Complement factors have been detected in substantial amounts within atherosclerotic plaques (Laine et al., 2002; Speidl et al., 2011a). Under normal conditions, activated complement components are quickly cleared from the circulation. However, activated complement and the MAC were identified within fatty streaks (early stages of plaque formation, before the arrival of inflammatory cells) in cholesterol-fed rabbits (Seifert et al., 1989). This finding was confirmed by following studies, which demonstrated the presence of the terminal complement complex C5b–9 in human atherosclerotic arteries (Niculescu et al., 1985, 1987; Torzewski et al., 1998).

**Figure 2:**
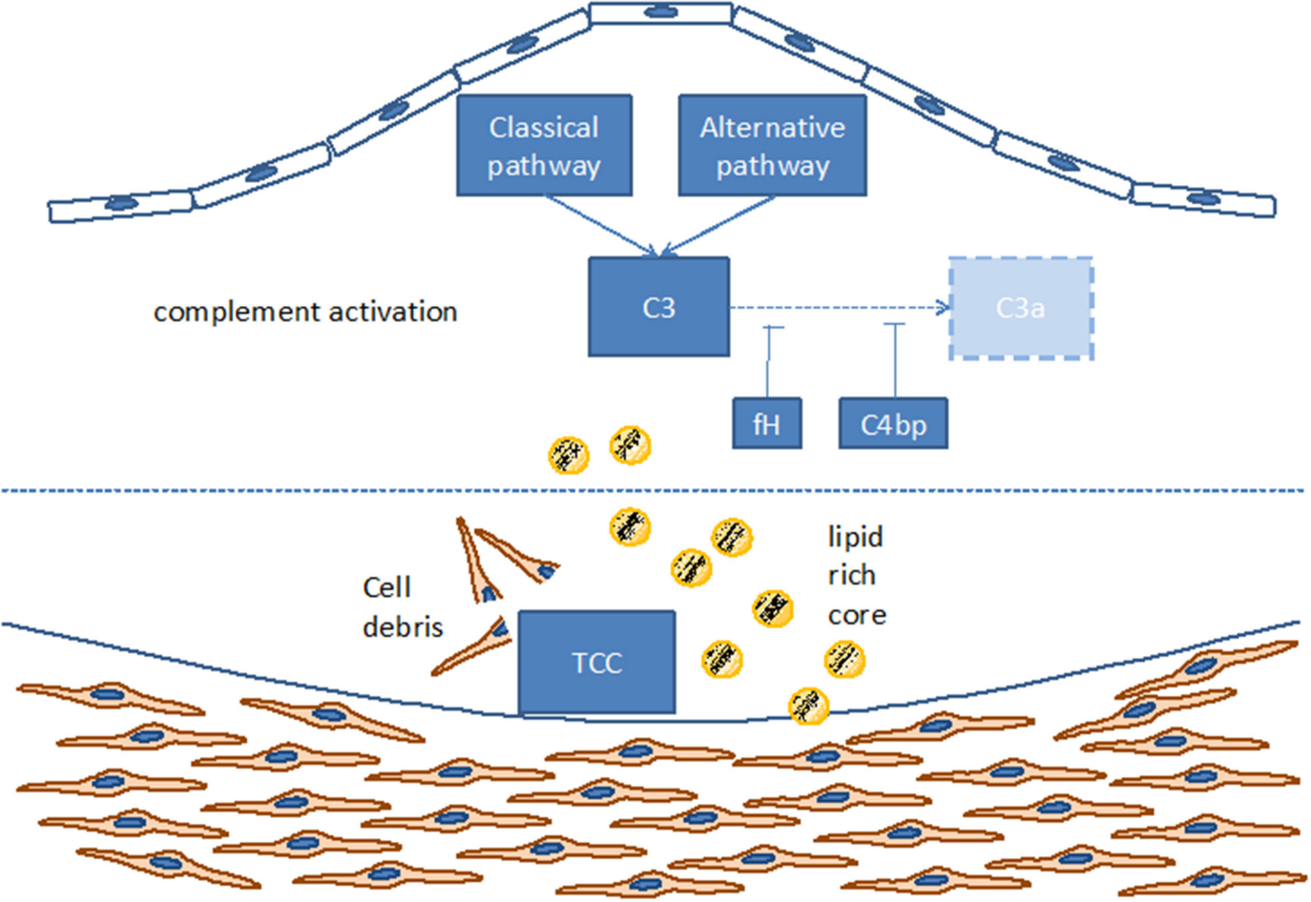
**Complement activation within the atherosclerotic plaque**. TCC, terminal complement complex; C3, C3a, C4bp, different components of the complement cascade; fH, factor H.

**Figure 3:**
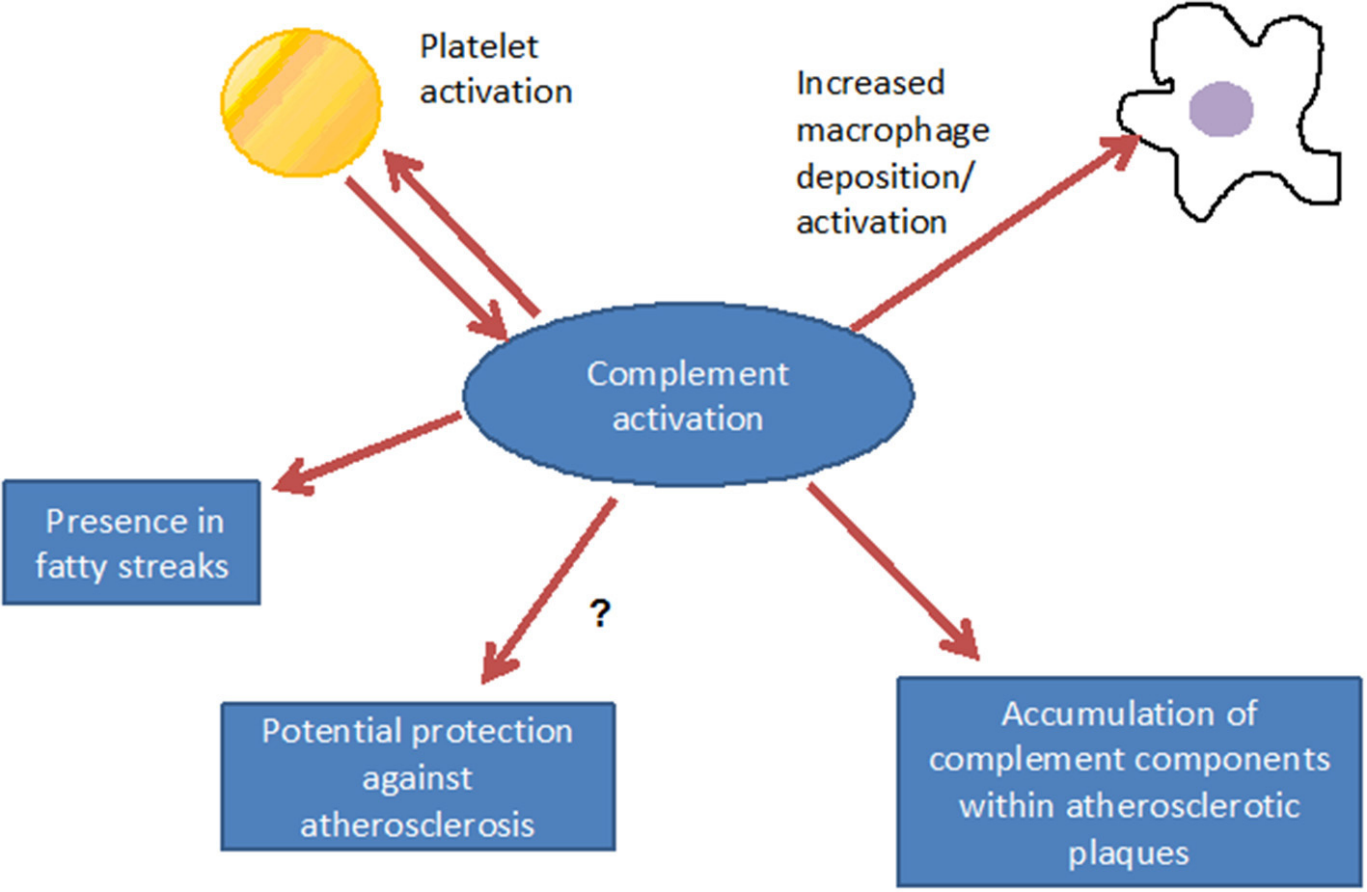
**Consequences of complement activation: complement interacts with inflammatory cells, may, however, also have a functional relationship with platelet activation**. Via promotion of inflammation, complement contributes to the formation of the fatty streak, under distinct conditions, however, atheroprotective effects can not be excluded.

Such complement components may derive from the blood circulation (Vlaicu et al., 1985; Niculescu and Rus, 2004), but the presence of mRNA for several complement components (including C1r, C1s, C4, C7, and C8) indicates that these factors also may be produced locally within the plaque (Yasojima et al., 2001; Niculescu and Rus, 2004). Indeed, various studies identified the power of local complement production (as opposed to circulating systemic complement) in driving immune processes (Verschoor et al., 2001, 2003; Gadjeva et al., 2002; Li et al., 2007). Interestingly, complement activation differs between superficial and deeper layers of the atherosclerotic plaque: in the luminal layer, signs of classical and alternative, but not terminal, complement activation can be found, consistent with the local presence of complement regulators C4bp and fH (Oksjoki et al., 2003, 2007). In contrast, terminal complement complex deposition is detected in the deeper layers of the intima, associated with smooth muscle cells, cell debris, and extracellular lipids (Oksjoki et al., 2003, 2007) (Figure 2). In addition, C1q and the receptor for its globular domain (gC1q-R) are found in the necrotic core of advanced atherosclerotic lesions (Peerschke et al., 2004). Also C3b can be detected, with stronger deposition in ruptured compared to non-ruptured plaques of the same patients (Laine et al., 2002). Another sign of increased complement activation within ruptured plaques is the significantly higher C5a concentration associated with lipid-rich inflammatory lesions containing exposed cholesterol and necrotic cell debris than with stable plaques containing collagen- and elastin (Speidl et al., 2011b). Epidemiological data underline this notion and show increased C5a levels in patients with increased cardiovascular disease risk, independent of non-specific inflammatory markers such as C-reactive protein (CRP) or fibrinogen (Speidl et al., 2005). Also elevated C4 levels in the circulation are associated with severe atherosclerosis (Muscari et al., 1988). Still, mere detection of complement activation or deposition does not yet ascertain a deleterious or a protective role for the complement system in artherosclerosis. In fact, various studies underline a pro-atherosclerotic role of complement, while others suggest protective effects for complement. Already in the late 1970s Geertinger *et al* could show that C6 deficiency, for instance, protects cholesterol-fed rabbits from atherosclerosis (Geertinger and Sorensen, 1970) and inhibition of C5a or its receptor C5aR1 (CD88) reduces atherosclerosis in murine models (Shagdarsuren et al., 2010; Manthey et al., 2011). On the other hand, C1q deficiency leads to the development of significantly larger lesions in atherosclerotic low-density lipoprotein receptor deficient (LDLR–/–) mice compared to C1q-sufficient controls (Bhatia et al., 2007; Lewis et al., 2009). One explanation for this observation may be that C1q binds apoptotic or necrotic cells in plaques directly (or indirectly via IgM), facilitating their removal by macrophages by promoting classical pathway deposition of C3 activation products. Indeed, C3 deficiency promotes the development of larger abdominal and thoracic aorta lesions in atherosclerotic LDLR–/– mice than in C3-sufficient controls. The aortic root lesions of the C3-deficient mice showed increased lipid and macrophage deposition, combined with decreased collagen and smooth muscle cell content, indeed suggesting a net protective effect for C3 in this model (Buono et al., 2002). This notion is underlined by the fact that mice lacking both ApoE and LDLR show a dramatically increased aortic lesions load (+84%) when C3 is absent too (Persson et al., 2004). Given the ambiguous experimental findings regarding the role of complement in the genesis of artherosclerosis, the efficacy of targeting complement in coronary artery disease (CAD) was directly assessed in several clinical trials. Consistent with the protective effects seen in experimental studies interfering with C5 function (Manthey et al., 2011; Shagdarsuren et al., 2010), treatment with anti-C5 antibody (Pexelizumab) resulted in significantly reduced mortality in ST-elevated myocardial infarction (STEMI) patients (Granger et al., 2003). Other trials that examined complement inhibition in patients undergoing coronary artery bypass graft surgery found positive effects on morbidity and mortality (Testa et al., 2011). All in all, a heterogeneous picture emerges regarding the role of complement in atherosclerosis and also open questions remain (Figure 3), which will have to be addressed in future basic and clinical studies to further define the role of complement and its potential and perspective for targeted treatment of atherosclerosis patients.

## Platelets and the complement system

Given the association of platelets and the complement system with the genesis and late complications of atherosclerosis, as well as their intimate contact in the circulation, a closer review of their interactions is warranted. A wide variety of complement factors and receptors have been identified in platelet isolates (Hamad et al., 2010). We recently found that the expression of anaphylatoxin-receptors (C3aR and C5aR) and activation markers (i.e., P-selectin) on platelets correlates with CAD in patients (Patzelt et al., 2014). Others described a potentially self-reinforcing cycle, in which complement can activate platelets and, in turn, thrombin activated platelets are able to initiate the complement cascade (Hamad et al., 2008). In addition, CR4, also a receptor for iC3b, is expressed on platelets but so far with unclear function (Vik and Fearon, 1987). Besides the aforementioned C3-fragment receptors, platelet expressed C1q receptors gC1qR/p33 and cC1qR were shown to mediate platelet aggregating and activating effects (Wautier et al., 1977; Peerschke and Ghebrehiwet, 1997, 2001). Also receptors for anaphylatoxins C3a and C5a, which are generated further downstream in the complement activation cascade, are found on platelets. C3a and its derivative C3a-des-Arg induce platelet activation and aggregation *in vitro* (Polley and Nachman, 1983; Martel et al., 2011).

The intimate contact between platelets and plasma proteins, including complement, combined with the propensity of platelets to become activated by complement, requires that platelets actively counteract complement deposition onto their surface. To this end, platelets express and bind a variety of complement control proteins (CCPs) on their membrane (Verschoor and Langer, 2013). Indeed, absence or impairment of function of such CCPs is associated with platelet dysfunction, and activation thrombocytopenia, again underlining the potential of the complement system to influence platelet physiology and function (Verschoor and Langer, 2013). In atypical hemolytic uremic syndrome (aHUS), deficiencies or mutations in CCPs, frequently in factor H, may result in excessive complement activation on platelets, leading to thrombocytopenia and prothrombotic complications (Stahl et al., 2008). Another immune mediated pathology in which platelets are affected by overwhelming complement activation is paroxysmal nocturnal hemoglobinuria (PNH). Here, a mutation in the phosphatidylinositol glycan A (PIGA) enzyme prevents the effective anchoring of CCPs decay-accelerating factor (DAF, CD55) and protectin (CD59) to the platelet surface (Nicholson-Weller et al., 1982). With the advent of Eculizumab, a humanized version of the anti-C5 antibody h5G1.1, which was first described in 1996, both conditions can now be effectively treated. Eculizumab is a drug approved for the treatment of PNH. By binding directly to C5, this mAb prevents the cleavage of C5–C5b and, thus, suppresses the formation of the membrane damaging MAC (Thomas et al., 1996).

Finally, platelets may also interact with the complement system via proteins that are not considered classical complement receptors, such as P-selectin (Del Conde et al., 2005) or GP1bα (Verschoor et al., 2011). In the case of P-selectin it was observed that it can bind C3b and mediate the generation of C3a and MAC formation (Del Conde et al., 2005). As exemplified by the pathology and treatment of aHUS and PNH, platelet activation is enhanced by MAC formation causing a prothrombotic state (Sims and Wiedmer, 1991). In the case of GPIbα, bacterial infection studies in mice revealed that, upon systemic infection, C3b-opsonized bacteria form complexes with platelets in the bloodstream. Such complexes can only form in the presence of the alpha chain of GPIb on the platelet surface, strongly suggesting that GPIb directly or indirectly interacts with activated complement C3 (Verschoor et al., 2011). Interestingly, both GPIb and C3 deficient mice show prolonged bleeding times (Strassel et al., 2007; Gushiken et al., 2009), strengthening the notion that these molecules may synergize in physiological hemostatic processes.

## Perspective and outlook

Various clinical and experimental lines of evidence indicate that platelets and the complement system influence the initiation and pathogenesis of atherogenesis and modulate each other's function. While clinical evidence is mounting, most of our mechanistic understanding of atherosclerosis still derives from animal models which model some, but not all characteristics of human atherosclerosis. With non-invasive methods lacking, our ability to directly assess the mechanisms, pathophysiology and plaque burden of human atherogenesis remains limited and indirect parameters (i.e., intima-media thickness, CT-based coronary calcium, mortality) have to be used instead. While such parameters are important in terms of practical patient care, it is often difficult to assess, whether i.e., anti-platelet treatment works by reducing atherosclerotic lesions or by preventing the thrombotic complications of plaque rupture. Thus, such studies unfortunately remain limited in helping us understand the underlying mechanisms. We therefore believe that, in addition, a hypothesis-driven approach is needed to bring seemingly disparate mechanisms in atherosclerotic lesion development together, as we attempt here in this review with complement and platelets.

Several studies show that activated complement is more abundant in unstable plaques than in stable atherosclerotic lesions. Underlining the notion that complement may have long remained an underestimated factor in artherosclerosis is provided by recent clinical studies that show that pexelizumab, a humanized monoclonal antibody against C5, clearly reduced rates of acute major cardiac events after STEMI or coronary bypass surgery (Granger et al., 2003; Verrier et al., 2004). Nonetheless, the drug failed to reduce mortality or recurrent myocardial infarction in the large, randomized controlled APEX AMI trial (Investigators et al., 2007).

Thus, the mixed results highlight once more the complexity of the pathological mechanisms underlying artherosclerosis and the need for intensified clinical and experimental studies probing the interaction of complement and platelets in the context of atherosclerosis. It stresses the need for a comprehensive approach, combining both clinical and experimental research, to uncover and detail further links between platelets and complement with the aim to identify new and promising pharmacological targets in the treatment of atherosclerosis.

### Conflict of interest statement

The authors declare that the research was conducted in the absence of any commercial or financial relationships that could be construed as a potential conflict of interest.
